# Use of Electro-Convulsive Therapy as a Bipolar Disorder Treatment: A Systematic Review

**DOI:** 10.1192/j.eurpsy.2022.1058

**Published:** 2022-09-01

**Authors:** E. Miranda Ruiz, E. Marimón Muñoz, A. Stoppa Montserrat, I. Fernandez, M.I. Arroyo, N. Borras, S. Ferreiro, J. Ramirez

**Affiliations:** 1 CST, Psychiatry, Terrassa, Spain; 2 CST, Psychiatry, Barcelona, Spain

**Keywords:** bipolar depression, bipolar disorder, Electro-convulsive therapy, mania

## Abstract

**Introduction:**

Electro-convulsive Therapy (ECT) has been considered a useful for the treatment of depression and other affective disorders, however it is considered as a last resort given the risks and possible adverse effects.

**Objectives:**

The objective of this review is to assess the use of ECT (in terms of efficacy and tolerability) for patients diagnosed with bipolar disorder and how it can be compared with other treatments more commonly used to treat this disorder.

**Methods:**

A search was carried out in Medline and in the Virtual Health Library as well as in the Trripdatabase with the search terms “Bipolar disorder”, “Bipolar Depression”, “ECT”, “ECT treatment” and “Mania” in English and narrowing the search to the last 5 years. 8 articles were included for the review after applying inclusion and exclusion criteria.

**Results:**

A favorable and well tolerated response was observed when applied ECT on patients with Bipolar disorder, especially the elderly populations. It was observed that the administration of unilateral and bilateral ECT are both equally effective. A better response was detected to ECT compared to newer treatments like ketamine, as well as lower suicide rate when ECT was used compared to other treatments.

**Conclusions:**

ECT is considered an effective and safe treatment for Bipolar Disorder and should be taken into account not only as a last resort. Even so, given the limitations observed, it is necessary to carry out further investigation on the matter.

**Disclosure:**

No significant relationships.

